# Patterns of care and outcome of *CIC*‐rearranged sarcoma patients: A nationwide study of the French sarcoma group

**DOI:** 10.1002/cam4.5539

**Published:** 2022-12-20

**Authors:** Mehdi Brahmi, Nathalie Gaspar, Justine Gantzer, Maud Toulmonde, Pascaline Boudou‐Rouquette, emmanuelle Bompas, Nelly Firmin, Thibaud Valentin, Mathilde Cancel, Florence Duffaud, Francois Bertucci, Christophe Perrin, Armelle Dufresne, Perrine Marec‐Bérard, Myriam Jean‐Denis, Isabelle Ray‐Coquard, Francois Le Loarer, Gaille Pierron, Franck Tirode, Jean‐Yves Blay, Sarah Watson

**Affiliations:** ^1^ Centre Léon Bérard Lyon France; ^2^ Gustave Roussy Cancer Campus Villejuif France; ^3^ ICANS Strabourg France; ^4^ Institut Bergonié Bordeaux France; ^5^ Hopital Cochin Saint Vincent de Paul Paris France; ^6^ ICO Angers France; ^7^ ICM Montpellier France; ^8^ Institut Claudius Regaud Toulouse France; ^9^ CHU Bretonneau Tours France; ^10^ CHU Timone Marseille France; ^11^ Institut Paoli‐Calmettes Marseille France; ^12^ Centre Eugène Marquis Rennes France; ^13^ Institut Curie Paris France

**Keywords:** CIC::DUX4 sarcomas, CIC‐rearranged sarcomas, Undifferentiated round cell sarcoma, Ultra‐rare sarcoma, Ewing‐like sarcoma.

## Abstract

**Background:**

*CIC‐*rearranged sarcomas (*CIC*‐RS) represent the most frequent subset of “Ewing‐like” undifferentiated small round cell sarcomas. These tumors tend to be more aggressive than Ewing sarcomas. Moreover, treatment strategy can differ according to teams. The primary aim of this retrospective study was to describe the characteristics, treatments, and outcome for patients with *CIC*‐RS included in the French NETSARC+ database.

**Methods:**

Pediatric and adult patients from 13 French centers with a diagnosis of *CIC*‐RS were registered from October 2008 to March 2021. Patients and tumors characteristics were collected from the national network NETSARC+ database (http://netsarc.sarcomabcb.org). *CIC*‐RS diagnosis was pathologically and molecularly confirmed with a central review by expert pathologists. Two groups of patients were studied: those treated as classical Ewing sarcomas (cohort EwS) and those treated as high‐grade soft tissue sarcomas (cohort STS) according to ESMO and/or EpSSG guidelines. Survival was calculated using the Kaplan–Meier method and the log‐rank test was used to compare survival.

**Results:**

Among 79 patients, the male/female sex ratio was 0.7 and the median age at diagnosis was 27 years (range 2–87). With a median follow‐up of 37 months, 39 patients died of the disease. Median overall survival from diagnosis was 18 months, with no significant difference between both cohorts (*p* = 0.9). Nevertheless, when focusing on patients with metastatic disease at diagnosis (*N* = 21), all patients from cohort STS died of disease while some patients from cohort EwS were still alive and in complete remission.

**Conclusion:**

FSG experience confirms the aggressive clinical course of CDS patients regardless of chemotherapy regimen.

## Graphical abstract

Two groups characterized by different patterns of care were evaluated in our study. On one hand (cohort 1), patients were treated as Ewing sarcomas, and on the other hand (cohort 2), patients were treated as high‐grade soft tissue sarcomas. Overall, the prognosis was poor and there were no statistical differences in overall survival and relapse‐free survival between the two groups. Nevertheless, a significant proportion of patients with metastatic CDS from cohort 1 achieved very long‐term survival and might be cured.

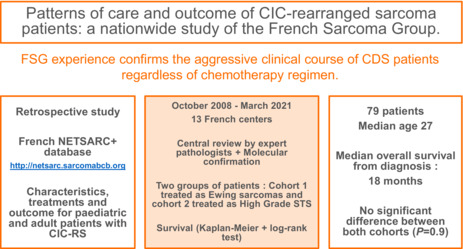

## 1 INTRODUCTION

Over the last decades, significant advances in the molecular refinement of small round blue cell sarcomas (SRBCS) have led to the identification of several new tumor entities, previously referred to as “Ewing‐like sarcomas” based on their clinical and pathological features showing partial overlap with Ewing sarcoma (EwS).^1^ Among them, three distinct sarcoma subtypes with specific clinical, pathological, and molecular features have been individualized in the last WHO classification, including *CIC*‐rearranged sarcoma (*CIC*‐RS), *BCOR*‐rearranged sarcoma, and SRBCS with *EWSR1*‐non *ETS* fusion.^2^

*CIC*‐RS consist in the most frequent subtype of these ultra‐rare tumors.^3^ They are composed of undifferentiated tumor cells arranged in solid sheets with frequent areas of necrosis and small to medium‐sized round nuclei and eosinophilic cytoplasm. Tumors cells classically show focal and patchy CD99 membranous staining, WT‐1 positivity, and ETV4 nuclear staining.^4^, ^5^, ^6^, ^7^ *CIC*‐RS are characterized by recurrent gene fusions involving *CIC* on chromosome 19, its most common fusion partner being *DUX4* on chromosome 4 or 10.^3^, ^4^ Alternative fusions of *CIC* with other partners including *NUMT1*, *NUTM2A*, *FOXO4*, *LEUTX*, and *CREBBP* have been reported.^8^, ^9^, ^10^, ^11^

From a clinical point of view, *CIC*‐RS present as aggressive tumors, most of the time developing from soft tissues and affecting young adults.^12^, ^13^, ^14^ In the largest series reported so far and including 57 *CIC*‐RS patients with clinical follow‐up available, the 5‐year overall survival (OS) rate was only 43%, significantly lower than in EwS.^12^ The management of such rare subtypes of sarcomas is challenging and most patients diagnosed over the last years have been treated according to EwS protocols.^15^ However, there is no consensus on whether they should be treated with an EwS approach or regarded as high‐grade STS.^16^ Therefore, treatment patterns widely differ according to teams and more studies are needed to address specifically the prognosis and management of *CIC*‐RS and to define their optimal management.

In this regard, the primary aim of this retrospective study was to describe characteristics, treatments, and outcome of patients with *CIC*‐RS included in the French NETSARC+ database.

## 2 MATERIALS AND METHODS

### 2.1 Patients

The study was conducted within NETSARC+, the French reference network for sarcomas. Data were collected within the national pediatric/adult sarcoma database (http://netsarc.sarcomabcb.org). Pediatric and adult patients with *CIC*‐RS diagnosed from October 2008 to March 2021 were analyzed. Pathological and molecular confirmation of the diagnosis by a central review from sarcoma expert pathologists of NETSARC+ network was mandatory. All cases were analyzed with RNA sequencing and/or RT‐PCR with specific combination of primer sets for *CIC::DUX4* and/or FISH analysis with probes for *CIC* (19q13.2). The study was first approved by the FSG (French Sarcoma Group) council the 2nd of December 2019, then declared to the *Commission Nationale de l'Informatique et des Libertés* (CNIL) and approved by the ethics committee. All living patients were informed of the study by mail and clinical data were updated on September 2021.

The following data were collected: age at diagnosis, sex, histology, location, depth, size, stage at diagnosis, treatment pattern, relapse, and survival. Two cohorts of patients were studied:

- **Cohort EwS** for patients treated according to standard treatment for EwS with multi‐agent chemotherapy (CT) before and after local therapy, with VIDE/VAC/VAI/VDC/IE (V = Vincristine, I = Ifosfamide, D = Doxorubicin, E = Etoposide, A = Actinomycin, C = Cyclophosphamide) and patients receiving Busulfan Melphalan (BuMel) High‐Dose CT. Patients receiving AI regimen (Doxorubicin ifosfamide) before local therapy and then VAI/VAC after local therapy were included in the cohort 1. Patients included in the EURO‐EWING 99 and 2012 trials (NCT00020566 and NCT00987636) were also included in cohort 1.
- **Cohort STS** for patients treated as high‐grade soft tissue sarcomas (HG‐STS) according to ESMO (European Society for Medical Oncology) or EpSSG (European Paediatric Soft tissue sarcoma Study Group) guidelines,^16^ mostly patients receiving three to five cycles of neoadjuvant AI, no adjuvant chemotherapy for localized disease, and anthracyclines‐based CT as the first‐line treatment for metastatic disease.

### 2.2 Statistical analysis

All clinical data were anonymized and retrospectively collected. Median relapse‐free survival (mRFS) and median overall survival (mOS) were calculated using the Kaplan–Meier method from the date of the initial diagnosis to the date of the occurrence of the reported event or the latest follow‐up, respectively. Significances are given by log‐rank tests, where *p* below 0.05 was considered as a significant survival difference between risk‐groups. These statistics were performed using R software.

## 3 RESULTS

### 3.1 Characteristics and treatment

In total, 79 patients with *CIC*‐RS were identified from 13 FSG centers. Their clinical and pathological characteristics are presented in Table 1. Five patients (6%) had a previous cancer history, including a neuroblastoma, an ovarian germ cell tumor and a breast cancer. Detailed information on treatment were available for 74 patients (94%), with 47 (59%) and 26 (33%) patients in cohort EwS and STS, respectively. One patient received a BEP (Bleomycin Etoposide Cisplatin) standard regimen for germ‐cell tumor and therefore was excluded from both cohorts. Furthermore, 45 patients (57%) received (neo)adjuvant radiotherapy with an median dose of 50 Grays [44–60]. Among cohort EwS, nine patients were enrolled in the EURO‐EWING 2012 trial. Comparing characteristics of patients in both cohorts, patients from cohort EwS were younger than in cohort STS (median age 21 [2–64] vs. 35 [4–87], *p* = 0.01) whereas there was no difference in median tumor size (100 mm [20–300] for cohort EwS and 96 mm [11–200] for cohort STS). In cohort EwS, 16 patients had metastatic disease at diagnosis (34%) versus 6 in cohort STS (23%) (*p* = 0.3).

**TABLE 1:** Clinical and pathological characteristics of the investigational series of CDS

	All series	Cohort EwS	Cohort STS
N	**79**	**47**	**26**
Sex ratio (male/female)	0.7 (33/46)	0.8 (21/26)	0.9 (12/14)
Median Age (years); range	27; 2–87	21; 2–64	35; 4–87
Number of pediatric patients (<18)	23 (29%)	18 (38%)	3 (12%)
Median size of the primary tumor (mm)	100 (11–300)	100 (20–300)	96 (11–200)
Primary tumor location			
Soft tissue	74 (94%)	42	26
Viscera	4 (5%)	4	0
Bone	1 (1%)	1	0
Stage at diagnosis			
Localized or locally advanced	57 (72%)	31 (64%)	20 (77%)
Metastatic	22 (28%)	16 (34%)	6 (23%)
Front‐line pattern of treatment			
EwS	47 (59%)		
HG‐STS	26 (33%)		
Germ cell tumor	1 (1%)		
N/A	5 (6%)		

In the front‐line setting, 22 patients received VIDE regimen (all form cohort EwS), 22 patients received VDC‐IE (all from cohort EwS), 23 patients received AI (three from cohort EwS and 20 from cohort STS), and 6 patients received Doxorubicin monotherapy (all from cohort STS). The response rates are detailed in Table 2, with an overall response rate of 54%, 46%, 30%, and 0 with VIDE, VDC/IE, AI, and Doxorubicin, respectively. Five patients received BuMel High‐Dose CT, among whom two were included in the EURO EWING 2012 trial.

**TABLE 2:** Response rate of the different front‐line regimens

Regimen/Response	CR	PR	SD	PD	NE	ORR
VIDE	2 (9%)	10 (45%)	5 (23%)	2 (9%)	3 (14%)	**54%**
VDC/IE	1 (5%)	9 (41%)	3 (14%)	2 (9%)	7 (32%)	**46%**
AIM	1 (4%)	6 (26%)	7 (30%)	3 (13%)	6 (26%)	**30%**
Doxorubicin	0	0	1 (17%)	1 (17%)	4 (66%)	**0**

Abbreviations: CR, complete response; NE, non evaluable; ORR, overall response rate; PD, progressive disease; PR, partial response; SD, stable disease.

In case of relapse/refractory disease, 26, 13, and 3 patients had access to second line, third line, and fourth line treatment respectively. They received a wide range of regimens, including irinotecan‐based chemotherapy (*N* = 12), pazopanib (*N* = 6), gemcitabine‐based chemotherapy (*N* = 5), topotecan‐cyclophosphamide (*N* = 3), trabectedin (*N* = 3), regorafenib (*N* = 3), anti‐PD1/PD‐L1 (*N* = 2), and carboplatin‐etoposide (*N* = 1). While most patients presented with a progressive disease as best response, three partial responses (with gemcitabine‐docetaxel, gemcitabine‐dacarbazine, and carboplatin‐etoposide) and two stable diseases (with irinotecan‐temozolomide and regorafenib) were achieved, as detailed in Table 3.

**TABLE 3:** Best response of the different regimens for relapse/refractory disease

Regimen / Best Response	Partial response	Stable disease	Progressive disease
Irinotecan based regimen (*N* = 12)	0	**1**	11
Gemcitabine based regimen (*N* = 5)	**2**	0	3
Topotecan Cyclophosphamide (*N* = 3)	0	0	3
Carboplatine Etoposide (*N* = 1)	**1**	0	0
Trabectedin (*N* = 3)	0	0	3
Pazopanib (*N* = 6)	0	0	6
Regorafenib (*N* = 3)	0	**1**	2
Anti‐PD1/PD‐L1 (*N* = 2)	0	0	2

### 3.2 Survival

**FIGURE 1:**
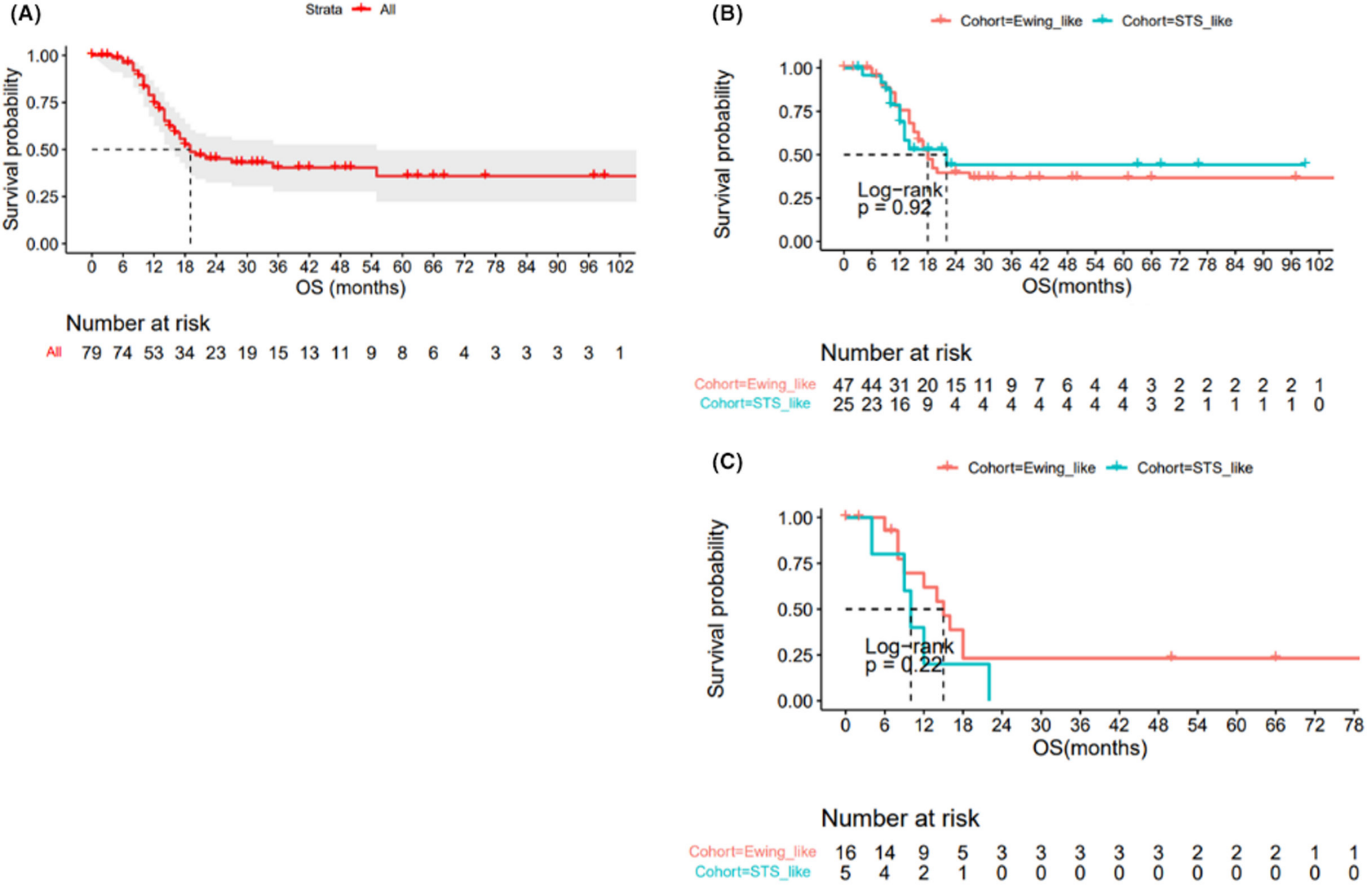
Overall survival of (A) whole cohort (B) whole cohort according to pattern treatment (C) patients with metastatic disease according to pattern of treatment.

After a median follow‐up of 37 months after diagnosis, 40 patients (51%) died, 39 from disease progression, and one from suicide. Median OS was 18 months (Figure 1A), with no significant difference between both cohorts (*p* = 0.92) (Figure 1B). When focusing on patients with localized disease at diagnosis, mRFS from diagnosis was 11 months, without any significant differences between cohort EwS and STS (*p* = 0.58). Importantly, when focusing on metastatic disease at diagnosis, all patients form cohort STS (5/5) died of disease, while some patients from cohort EwS were still alive (5/16), leading to a long‐term survival plateau (median OS 10 vs. 15 months, *p* = 0.22; Figure 1C). All those five patients presented with primary lung‐only metastases and achieved a radiologic complete response on the chest CT (Computed tomography) scan after neoadjuvant/adjuvant chemotherapy.

## 4 DISCUSSION

*CIC*‐RS is an ultra‐rare subtype of sarcoma.^17^ Literature about this disease remains scarce and most of the studies reported so far include case reports or small series. Antonescu et al. reported the clinical follow‐up of 57 patients with a 5‐year OS of 43%, which was significantly lower than the 77% 5‐year OS of the EwS control group (*p* = 0.002).^12^ Another series of 18 patients reported a median OS of 16 months and a median duration of systemic treatment response of 2 months.^11^ To our knowledge, with 79 patients included, this French multi‐institutional retrospective study provides the largest cohort so far, to describe not only the characteristics and outcomes but also the patterns of care of a molecularly defined series of *CIC*‐RS. It confirms the highly aggressive clinical course of *CIC*‐RS patients, with a median OS of 18 months, consistent with the recent literature.

As of today, the scientific community still aims at identifying the best therapeutic strategy for *CIC*‐RS patients. *CIC*‐RS consistently appear to be less chemo‐sensitive than EwS in the up‐front setting.^18^ However, our study shows that multi‐agent regimens can achieved a good response rate (up to 54%), even though duration of response remains limited. Furthermore, the literature analysis reported several cases of good response to chemotherapy according to standard protocols applied to EwS.^19^, ^20^ Depending on the initial therapeutic strategy, two groups of CRS were evaluated in the study: those treated as EwS and those treated as HG‐STS. There was no statistical difference in mOS and mRFS between both cohorts. Nevertheless, when focusing on metastatic disease at diagnosis, long‐term survivors were only observed in cohort EwS, with an interesting long‐term survival plateau. Previously, Smith et al.^21^ reported a cohort of 10 cases of *CIC*‐RS patients, including two metastatic cases with prolonged survival (no evidence of disease at 22 and 48 months respectively) after EwS regimen. Therefore, our study suggest that dose‐intensive chemotherapy might offer some benefit in selected patients and metastatic CRS should not be considered an incurable disease. Otherwise, *CIC*‐RS patients with recurrent/refractory disease have limited therapeutic options. In this series, while almost all treatments resulted in PD as best response, two PR were reported with gemcitabine‐based regimens, which could therefore represent a therapeutic option. Anyway, the prognosis remains very poor and inclusion of *CIC*‐RS patients in clinical trials should be encouraged. Importantly, patients with recurrent/refractory *CIC*‐RS can be enrolled in a phase II trial (NCT02389244) which evaluates the efficacy of regorafenib in this population.

Our series has several limitations. First, data are retrospective. Second, other classical prognostic factors such as biological parameters,^22^ tumors characteristics (for instance number and site of metastasis), and complementary therapeutic management (such as palliative care) were not available for all patients. Finally, because of the rarity and the aggressiveness of the disease, our series included few long‐term survivor patients, responsible of low power study. What should be the next steps? A longer follow‐up and above all an international collaboration are crucial. Conducting randomized studies to compare the efficacy of different regimens could also be of interest, though it could be challenging in this ultra‐rare disease.

In conclusion, the FSG experience confirms the poor prognosis of *CIC*‐RS regardless of the pattern of treatment. Even if the best therapeutic strategy remains currently elusive, this study suggests that patients should not benefit from a therapeutic de‐escalation, especially in case of metastatic disease. In is a useful support for future clinical trials and subsequent studies. It encourages new therapeutics and confirms that translational ancillary studies are capital.

## AUTHOR CONTRIBUTIONS

**Mehdi Brahmi:** Conceptualization (equal); data curation (equal); formal analysis (equal); investigation (equal); methodology (equal); project administration (equal); resources (equal); software (equal); supervision (equal); validation (equal); visualization (equal); writing – original draft (equal). **NATHALIE GASPAR:** Investigation (equal); resources (equal); writing – review and editing (equal). **Justine Gantzer:** Resources (equal); writing – review and editing (equal). **Maud Toulmonde:** Investigation (equal); writing – review and editing (equal). **Pascaline Boudou‐Rouquette:** Resources (supporting). **emmanuelle bompas:** Data curation (equal); visualization (equal). **Nelly Firmin:** Resources (supporting). **Thibaud Valentin:** Resources (supporting). **Mathilde Cancel:** Validation (equal). **florence duffaud:** Resources (equal). **Francois Bertucci:** Writing – review and editing (equal). **Christophe Perrin:** Resources (supporting). **Armelle Dufresne:** Validation (equal); writing – review and editing (equal). **Perrine Marec‐berard:** Resources (equal). **Myriam Jean‐Denis:** Software (equal). **isabelle ray‐coquard:** Visualization (equal). **François Le Loarer:** Formal analysis (equal). **Gaëlle Pierron:** Formal analysis (equal). **Franck Tirode:** Data curation (equal). **Jean‐Yves Blay:** Funding acquisition (equal); investigation (equal); methodology (equal); supervision (equal); validation (equal); visualization (equal); writing – review and editing (equal). **Sarah Watson:** Methodology (equal); validation (equal); visualization (equal); writing – original draft (equal); writing – review and editing (equal).

## FUNDING INFORMATION

LYRIC‐INCA DGOS4664, Lyrican INCa_INSERM_DGOS_12563, InterSARC grants, Infosarcome.

## CONFLICT OF INTEREST

MB declares honoraria from Bayer and Amgen and Accommodations, Expenses from PharmaMar; AD declares honoraria from Bayer and research support from GSK; JYB declares research honoraria from Novartis, GSK, Bayer, Roche, Deciphera, Ignyta, BMS, MSD, Pharmamar, Karyopharm and Research Support from Novartis, GSK, Bayer, Roche, Deciphera, Ignyta, BMS, MSD, Pharmamar, Karyopharm; other authors declare no potential conflict of interest.

## Data Availability

Data sharing is not applicable to this article as no new data were created or analyzed in this study.

## References

[1] Watson S , Perrin V , Guillemot D , et al. Transcriptomic definition of molecular subgroups of small round cell sarcomas. J Pathol. 2018;245(1):29‐40. 29431183 10.1002/path.5053

[2] WHO Classification of Tumours Editorial Board . Soft tissue and Bone Tumours. Vol 3. 5th ed. International Agency for Research on Cancer; 2020:607.

[3] Yoshimoto T , Tanaka M , Homme M , et al. CIC‐DUX4 induces small round cell sarcomas distinct from Ewing sarcoma. Cancer Res. 2017;77(11):2927‐2937. 28404587 10.1158/0008-5472.CAN-16-3351 PMC5488331

[4] Specht K , Sung YS , Zhang L , Richter GHS , Fletcher CD , Antonescu CR . Distinct transcriptional signature and immunoprofile of CIC‐DUX4 fusion‐positive round cell tumors compared to EWSR1‐rearranged Ewing sarcomas: further evidence toward distinct pathologic entities. Genes Chromosomes Cancer. 2014;53(7):622‐633. 24723486 10.1002/gcc.22172 PMC4108073

[5] Yamada Y , Kuda M , Kohashi K , et al. Histological and immunohistochemical characteristics of undifferentiated small round cell sarcomas associated with CIC‐DUX4 and BCOR‐CCNB3 fusion genes. Virchows Arch. 2017;470(4):373‐380. 28197724 10.1007/s00428-017-2072-8

[6] Le Guellec S , Velasco V , Pérot G , Watson S , Tirode F , Coindre JM . ETV4 is a useful marker for the diagnosis of CIC‐rearranged undifferentiated round‐cell sarcomas: a study of 127 cases including mimicking lesions. Mod Pathol. 2016;29(12):1523‐1531. 27562494 10.1038/modpathol.2016.155

[7] Kawamura‐Saito M , Yamazaki Y , Kaneko K , et al. Fusion between CIC and DUX4 up‐regulates PEA3 family genes in Ewing‐like sarcomas with t(4;19)(q35;q13) translocation. Hum Mol Genet. 2006;15(13):2125‐2137. 16717057 10.1093/hmg/ddl136

[8] Le Loarer F , Pissaloux D , Watson S , et al. Clinicopathologic features of CIC‐NUTM1 sarcomas, a new molecular variant of the family of CIC‐fused sarcomas. Am J Surg Pathol. 2019;43(2):268‐276. 30407212 10.1097/PAS.0000000000001187

[9] Sugita S , Arai Y , Aoyama T , et al. NUTM2A‐CIC fusion small round cell sarcoma: a genetically distinct variant of CIC‐rearranged sarcoma. Hum Pathol. 2017;65:225‐230. 28188754 10.1016/j.humpath.2017.01.012

[10] Sugita S , Arai Y , Tonooka A , et al. A novel CIC‐FOXO4 gene fusion in undifferentiated small round cell sarcoma: a genetically distinct variant of Ewing‐like sarcoma. Am J Surg Pathol. 2014;38(11):1571‐1576. 25007147 10.1097/PAS.0000000000000286

[11] Connolly EA , Bhadri VA , Wake J , et al. Systemic treatments and outcomes in CIC‐rearranged sarcoma: a national multi‐Centre clinicopathological series and literature review. Cancer Med. 2022;11(8):1805‐1816. 35178869 10.1002/cam4.4580 PMC9041083

[12] Antonescu CR , Owosho AA , Zhang L , et al. Sarcomas with CIC‐rearrangements are a distinct pathologic entity with aggressive outcome: a Clinicopathologic and molecular study of 115 cases. Am J Surg Pathol. 2017;41(7):941‐949. 28346326 10.1097/PAS.0000000000000846 PMC5468475

[13] Yoshida A , Goto K , Kodaira M , et al. CIC‐rearranged sarcomas: a study of 20 cases and comparisons with Ewing sarcomas. Am J Surg Pathol. 2016;40(3):313‐323. 26685084 10.1097/PAS.0000000000000570

[14] Gambarotti M , Benini S , Gamberi G , et al. CIC‐DUX4 fusion‐positive round‐cell sarcomas of soft tissue and bone: a single‐institution morphological and molecular analysis of seven cases. Histopathology. 2016;69(4):624‐634. 27079694 10.1111/his.12985

[15] Strauss SJ , Frezza AM , Abecassis N , et al. Bone sarcomas: ESMO‐EURACAN‐GENTURIS‐ERN PaedCan clinical practice guideline for diagnosis, treatment and follow‐up. Ann Oncol. 2021;32(12):1520‐1536. 34500044 10.1016/j.annonc.2021.08.1995

[16] Gronchi A , Miah AB , Dei Tos AP , et al. Soft tissue and visceral sarcomas: ESMO‐EURACAN‐GENTURIS clinical practice guidelines for diagnosis, treatment and follow‐up☆. Ann Oncol. 2021;32(11):1348‐1365. 34303806 10.1016/j.annonc.2021.07.006

[17] Stacchiotti S , Frezza AM , Blay JY , et al. Ultra‐rare sarcomas: a consensus paper from the connective tissue oncology society community of experts on the incidence threshold and the list of entities. Cancer. 2021;127(16):2934‐2942. 33910263 10.1002/cncr.33618 PMC8319065

[18] Renzi S , Anderson ND , Light N , Gupta A . Ewing‐like sarcoma: an emerging family of round cell sarcomas. J Cell Physiol. 2019;234(6):7999‐8007. 30257034 10.1002/jcp.27558

[19] Donthi D , Malik P , Prenshaw KL , Hong H . A rare case of round cell sarcoma with CIC‐DUX4 mutation mimicking a Phlegmon: review of literature. Am J Case Rep. 2020;21:e925683. 32873768 10.12659/AJCR.925683 PMC7491946

[20] Vieira AC , Xavier CB , Vieira TD , et al. CIC‐DUX4 rearranged uterine cervix round‐cell sarcoma exhibiting near‐complete pathologic response following radiation and neoadjuvant chemotherapy: a case report. Gynecol Oncol Rep. 2021;36:100745. 33850994 10.1016/j.gore.2021.100745 PMC8022141

[21] Smith SC , Buehler D , Choi EYK , et al. CIC‐DUX sarcomas demonstrate frequent MYC amplification and ETS‐family transcription factor expression. Mod Pathol. 2015;28(1):57‐68. 24947144 10.1038/modpathol.2014.83

[22] García‐Ortega DY , Melendez‐Fernandez AP , Alvarez‐Cano A , et al. Neutrophil‐to‐lymphocyte ratio as a prognostic biomarker in extremities undifferentiated pleomorphic sarcoma. Surg Oncol. 2022;42:101746. 35378375 10.1016/j.suronc.2022.101746

